# Supplementary material for: Learning curve and surgical outcome of robotic assisted colorectal surgery with ERAS program
Source: Sci Rep. 2022 Nov 29;12:20566. doi: 10.1038/s41598-022-24665-w (PMC9709162; doi:10.1038/s41598-022-24665-w)
Supplement: Supplementary file 1 — Supplementary Information 1. [file 41598_2022_24665_MOESM1_ESM.zip › LSC Raw data-Ñ[▒K/053/P_ñw╝╨░O▒Kñσ.pdf]

臺中榮民總醫院 臺中市臺灣大道四段1650號 病理部活體組織切片報告 臨床醫學實驗及病理診斷中心 檢驗(查)報告

[redacted]

Pathologic diagnosis:
1. Ascending, transverse, and descending colon, terminal ileum, and appendix, extended right hemicolectomy --- a. Adenocarcinoma, moderately to poorly differentiated, of transverse colon, with extracellular mucin production. Tumor invades through the visceral peritoneum, and metastasizes to mesocolic lymph node (1/32). The appendix, ileocecal valve, and bilateral cut ends are free of tumor.
b. Status post loop colostomy with stitch granuloma of mesocolon.
2. Gallbladder, cholecystectomy --- Cholesterolosis.

已電子簽章
警告：本單僅供醫師參考使用，不具任何申請效力！
請病患妥為保管，若不慎遺失需自負個人資料外洩責任！

Ancillary study for diagnosis:
IHC stains for MLH1, MSH2, PMS2 and MSH6 are done on section G. All above markers are preserved.

Prognostic and predictive factor:
1. Tumor size: 7.2x6.2x2 cm.
2. Depth of invasion: Visceral peritoneum (including tumor continuous with serosal surface through area of inflammation).
3. Histologic grade: G2-3: Moderately to poorly differentiated.
4. Angiolymphatic invasion: Not identified.
5. Perineural invasion: Not identified.
6. Tumor deposits (discontinuous extramural extension): Not identified.
7. Surgical margins:
a) Proximal Margin: Uninvolved by invasive carcinoma, distance of tumor from margin: more than 100 mm.
b) Distal Margin: Uninvolved by invasive carcinoma, distance of tumor from margin: 110 mm.
c) Radial or Mesenteric Margin: Uninvolved by invasive carcinoma, distance of tumor from margin: more than 10 mm.
8. Lymph node status: Involved (1/32).
9. Extranodal involvement: Not identified.
10. Tumor regression grading S/P CCRT: No known presurgical therapy.
11. Type of polyp in which invasive carcinoma arose: Not identified.
12. Additional pathologic findings: Not identified.
13. Pathological TNM Stage: pT4aN1a (According to the eighth edition, 2017, American Joint Committee on Cancer Staging Guidelines for Tumors / 2017 CAP guideline).
14. TNM descriptors: Not applicable.

Gross description:
The specimen consists of 1) a segment of colon, 41 cm in length, and 4 cm in proximal circumference, 6 cm in distal cut end circumference, and a segment of terminal ileum, 3.3 cm in length, and 4 cm in cut end circumference. An unremarkable appendix, 3.8 cm in length, is noted. A gray tan firm annular elevated and centrally ulcerative tumor, 7.2x6.2x2 cm, is noted, 11 cm to the distal cut end. On cross section, the tumor invades to the mesocolon with more than 10 mm to the radial margin and less than 1 mm to the serosa grossly. Serosal retraction is also noted focally. The appendix, ileocecal valve, and both cut ends are free of the tumor. 2) an opened gallbladder, a piece, 9x3.2x1.2 cm. The wall is gray tan soft and up to 0.3 cm in thickness. Neither stone nor tumor is found grossly. Representative part for section: A) gallbladder B) proximal cut end C) distal cut end D) appendix E) ileocecal valve F-K) tumor L-N) lymph nodes.

Note:
1. The interpretation of MMR panel and recommended studies are as below: (1) No loss of nuclear expression of MMR proteins: low probability of microsatellite instability-high (MSI-H). (2) Please note: There are exceptions to the above IHC interpretations. These results should not be considered in isolation, and clinical correlation with genetic counseling is recommended to assess the need for germline testing.
2. This case has been peer reviewed by two doctors.
#T-59441_2 #M-81403_2 3 1105 S0000F
#T-C4520_2 #M-81406_2 3 1105 000000
#T-59201_2 #M-09413_2 0 1105 000000
